# Sleepless behind bars: the connection between mental health, environment, and sleep among women in jail

**DOI:** 10.1093/sleepadvances/zpae012

**Published:** 2024-02-14

**Authors:** Emma J Tussey, Gabriela R Perez, Shannon M Lynch

**Affiliations:** Department of Psychology, Idaho State University, Pocatello, ID, USA; Department of Psychology, Idaho State University, Pocatello, ID, USA; Department of Psychology, Idaho State University, Pocatello, ID, USA

**Keywords:** insomnia, sleep quality, jail, depression, posttraumatic stress disorder

## Abstract

**Study Objectives:**

Given the barriers to good sleep in corrections facilities and the lack of research in this area, the current study aimed to characterize sleep quality and insomnia incidence in women in jail. Furthermore, we aimed to investigate the relation of sleep to depression, posttraumatic stress disorder (PTSD), and trauma exposure in incarcerated women. Lastly, we examined self-reports of environmental and individual factors that impaired sleeping in this population.

**Methods:**

Participants included 176 women incarcerated in two jails in southeast Idaho. Participants were randomly selected to complete several self-report questionnaires, including the Pittsburgh Sleep Quality Index and the Insomnia Severity Index, verbally administered by interviewers.

**Results:**

A majority of participants endorsed poor sleep quality (76%) and subthreshold or higher levels of insomnia (70%). Multiple regression analyses found that depressive symptoms and PTSD symptoms were both significantly related to insomnia and poor sleep quality. Excessive noise, poor bedding, and mental health were commonly cited factors that disrupted sleep.

**Conclusions:**

These results are consistent with previous literature that has examined these outcomes in prison populations and incarcerated populations in other countries. Correctional facilities can consider altering environmental factors that impair sleep to not only promote better overall health but also as a way to address common manifestations of poor mental health in their inmate populations. Screening for and treating mental health problems, namely depression and PTSD, is another way these facilities can improve inmate health and promote better sleep.

**Statement of Significance**

Our findings provide insight into the prevalence of sleep disturbance amongst women in short-term incarceration settings (e.g. jail). Women in jail experienced poor sleep quality, insomnia symptoms, and short sleep duration at greater levels than the general population. Our findings also illustrate potential causes of sleep disturbance in jail. Depression and posttraumatic stress disorder symptoms both correlated with poor sleep quality and insomnia in jail. Participants also indicated a number of environmental factors in jail that impacted their sleep (e.g. noise, lights, poor bedding, and trouble accessing medication). Overall, these findings describe the problem of sleep disturbance in jail and provide insight into how sleep could be improved in this setting. This information is critical to promoting health in this vulnerable population.

## Introduction

It is critical to examine factors that influence health outcomes in incarcerated women given the high early mortality rates in this population [1, 2]. Women who have experienced incarceration are over five times more likely to experience premature mortality relative to women who have not experienced incarceration [1]. One potential risk factor for early mortality in this population is sleep disturbance. There is robust evidence that sleep disturbance negatively impacts physical and psychological health outcomes [3, 4]. The few studies that have been conducted on sleep in incarcerated populations indicate that inmates experience high levels of insomnia and poor sleep quality [5–8]. However, there is still relatively little research on the sleep health of women in this population. Examining sleep in incarcerated women is imperative as prior research demonstrates that women experience higher rates of insomnia than men [9]. In addition, most studies assessing sleep in incarcerated individuals have been conducted in long-term correctional facilities (e.g. prisons) outside the United States [5–8]. Short-term incarceration facilities (e.g. jails) tend to be more overcrowded, with high turnover, and inmates often do not have their own assigned sleeping space like they might in prisons. More research is needed to characterize sleep health in women incarcerated in the United States, especially in shorter-term facilities (e.g. jails).

Correctional facilities pose numerous environmental challenges to good sleep. Considerable litigation has been brought against correctional facilities for conditions that impact inmates’ ability to sleep [10]. These lawsuits mention conditions such as constant noise and light, inadequate bedding, inability to access sleep medications, early wake times, and policies that inhibit quiet hours [10]. Some of these lawsuits suggest that conditions that prohibit sleep constitute cruel and unusual punishment for inmates, as sleep is a basic human need. Studies have shown that sleep is highly disturbed in incarcerated populations. A recent systematic review estimated that 26.2% to 72.5% of inmates had insomnia, and 42.8% to 88.2% of inmates had poor sleep quality [5]. In comparison, estimates for the general population include a 6%–30% [5, 11, 12] prevalence of insomnia and a 25% to 36% prevalence of poor sleep quality [5, 11, 13]. It is essential to understand why rates of insomnia and poor sleep quality are higher in incarcerated individuals.

The Spielman 3P model of insomnia suggests that insomnia develops based on an interaction between predisposing, precipitating, and perpetuating factors [14, 15]. Predisposing factors are biopsychosocial risk factors, which place some individuals at a higher probability of developing insomnia [15, 16]. Precipitating factors are acute occurrences that trigger sleep disturbance, for instance, an exposure to a traumatic event, psychiatric illness, or sleeping in a new environment [16, 17]. Perpetuating factors are the actions that an individual experiencing insomnia engages in which sustain insomnia over time [16]. These include learned associations, behaviors, and thought patterns, which inhibit sleep [18]. In addition to environmental risk factors, incarcerated individuals may be at higher risk of experiencing insomnia and sleep disturbance than the general population due to psychiatric risk factors which precipitate and perpetuate insomnia.

Incarcerated women exhibit higher prevalence rates for most psychological disorders than women in the general population [19]. Around half of incarcerated women meet diagnostic criteria for posttraumatic stress disorder (PTSD) in their lifetimes, compared to under 10% of women in the general population [20, 21]. The lifetime prevalence of major depressive disorder is also elevated in incarcerated women [21], compared to the general population [22]. For example, in a study of 491 incarcerated women across nine jails in the United States, the lifetime prevalence rate for major depressive disorder was 28%, compared to a lifetime rate of 10.4% for women in the general population [21, 22].

The high prevalence of psychological disorders amongst incarcerated women may be a contributing factor to the high rates of insomnia and poor sleep quality in this population, given that subjective and objective sleep disruption are hallmark symptoms of almost all psychiatric disorders [23–25]. Research suggests that this is a bidirectional relationship, such that psychiatric conditions can lead to sleep disturbance and that sleep disturbance can be a risk factor for psychiatric disorders [25, 26]. A recent systematic review summarized the literature on within-person associations between PTSD symptoms and sleep quality [27]. This review indicated that sleep disturbance predicts greater next-day PTSD symptoms and that PTSD symptoms predicted greater same-night sleep disturbance, thus demonstrating a bidirectional relationship. Therefore, it is essential to consider how psychiatric conditions contribute to insomnia in jail and how incarceration worsens sleep disturbance and psychiatric symptoms.

The main scope of the present cross-sectional study is to characterize sleep quality and the incidence of clinically significant insomnia symptoms in women jailed in the United States. We hypothesized that incarcerated women would have high rates of insomnia symptoms and poor sleep quality compared to the general population. To explore this, we observed similarities and differences between the prevalence of poor sleep quality and insomnia symptoms from 176 women in jail and published rates from the general population. Additionally, we aimed to examine relations among psychological distress, insomnia symptoms, and sleep quality in this population. We examined this through self-reports of depressive symptoms, PTSD symptoms, and frequency of trauma exposure. We hypothesized that greater trauma history, PTSD symptoms, and depressive symptoms would predict more insomnia symptoms and poorer sleep quality. Lastly, we were interested in environmental and individual factors that may worsen sleep quality in jail. We examined these factors by asking women to describe the reasons why they had difficulty sleeping in jail.

## Materials and Methods

### Participants

We randomly selected participants from two jails in southeast Idaho. Sleeping arrangements among the jails were similar in frequency of nightly checks by corrections officers and bedding provided. One facility utilized a communal sleeping space with bunk beds in each pod, while the other had shared cells and communal sleeping areas. Participants were eligible to participate if they identified as women, were at least 18 years old, and were fluent in English. We conducted a power analysis for our proposed analyses based on six predictors, a power of.90 and a moderate effect size (*f*^2^ ≥.15). This analysis indicated we needed a sample of 123 to achieve a power of 0.90. A total of 180 incarcerated individuals participated in the study. Individuals who did not identify as women (*n* = 4) were removed from analyses, resulting in a current sample of 176.

The participants’ ages ranged from 18 to 63 years old (*M* = 35.15, *SD* = 9.22). Most women identified as white/European-American (60.2%), while 14.2% identified as Native American/American Indian, 11.9% identified as Hispanic or Latina, and 11.0% identified as biracial or multiracial. On average, women were incarcerated for 68 days at the time of their interview (*SD* = 92.12 days), with all but two participants reporting incarceration periods between 1 day and 12 months. The majority of women (84.1%) reported prior detentions in jail. The most common charges (44.3%) were possession of illicit substances or paraphernalia. The next most common charges included parole or probation violations (27.3%), manufacturing, selling, or intent to sell illicit substances (8.0%), and assault or battery (6.8%).

### Procedures

The study design, variables, analyses, and hypotheses were preregistered on the Open Science Framework in August 2023. Study procedures were approved by the university Institutional Review Board. A federal certificate of confidentiality was obtained from the National Institutes of Health before data collection to protect the disclosure of sensitive research information. Names of current inmates were entered into a random number generator to create a list of potential participants every 2 weeks. The names were provided in the specified order to corrections officers who notified women they were selected and invited to participate in a study. Memos were also posted in the common areas to alert women that a study was being conducted and that they had the right to participate or decline with no negative consequences. Women who accepted the invitation reviewed the purpose of the study and then the consent form with the interviewer. To address variable reading levels among the participants, interviewees received a printed copy of the consent and questionnaires to review while trained interviewers verbally administered all questionnaires and documented answers given by the participants. Participants were compensated with a candy bar. A total of 25 women declined to participate.

### Measures

#### Demographics.

We collected demographic information from participants, including age, race/ethnicity, gender identity, and legal history.

#### Life Stressors Checklist-Revised.

The Life Stressors Checklist-Revised (LSC-R) is a 30-item measure of a broad range of traumatic experiences, including interpersonal trauma, accidents, natural disasters, and other significant adverse experiences [28]. We adapted the original response options to assess cumulative experiences of trauma. Instead of the presence or absence of an experience, respondents indicated the frequency of each experience, ranging from never to more than five times. Furthermore, items regarding interpersonal violence exposure were asked for childhood and adulthood, as opposed to the original items of lifetime interpersonal violence. Other studies have utilized these adaptations with incarcerated women in particular [29, 30]. Total scores range from 0 to 168, with higher scores representing more lifetime trauma exposure.

#### Posttraumatic Stress Disorder Checklist for the DSM-5.

The Posttraumatic Stress Disorder Checklist for the DSM-5 (PCL-5) is a 20-item evaluation of PTSD symptoms in the past month [31]. A total score is created by summing all items for scores ranging from 0 to 80, with higher scores indicating more severe PTSD symptoms and scores above 33 indicating a probable PTSD diagnosis [31]. This is a widely used and validated measure of PTSD symptoms that demonstrated excellent reliability in the present study (Cronbach’s *α* = .94). The PCL-5 was administered immediately following the LSC-R, and participants were instructed to answer the PCL-5 items with reference to traumatic events that were reported on the LSC-R. We removed one item that referenced sleep from the PCL-5 scoring when comparing PTSD to insomnia and sleep quality. Descriptive scores were calculated using the full measure.

#### Center for Epidemiological Studies Depression Scale Revised.

The Center for Epidemiological Studies Depression Scale Revised (CESD-R) is a 20-item measure of depressive symptom severity in the past 2 weeks [32]. A total score is created by summing responses from all items with scores ranging from 0 to 60. Higher scores are indicative of greater frequency of depressive symptoms, and scores equal to or greater than 16 are indicative of clinically significant symptoms of depression. The CESD-R exhibited excellent reliability in the current study (Cronbach’s *α* = .93). We removed three items that referenced sleep from the CESD-R scoring when comparing depression to insomnia and sleep quality. Descriptive scores were calculated using the full measure.

#### Pittsburgh Sleep Quality Index.

The Pittsburgh Sleep Quality Index (PSQI) is a 19-item questionnaire that measures global sleep quality over the past month [27, 33]. We modified this questionnaire for individuals who were incarcerated for less than one month and asked them to reflect on their sleep quality since they were incarcerated. The PSQI includes seven component scores that measure sleep duration, sleep disturbance, sleep latency, habitual sleep efficiency, use of sleep medications and daytime dysfunction, and overall sleep quality. These component scores are summed to create a global sleep quality score. Higher scores indicate poorer sleep quality. The PSQI demonstrated acceptable reliability in this study (Cronbach’s *α* = .64). The PSQI also includes a qualitative question that asks individuals to describe reasons they are having difficulty sleeping. Answers to this qualitative question were coded and presented descriptively to indicate internal and environmental factors that may lead to sleep disturbance in jail.

#### Insomnia Severity Index.

The Insomnia Severity Index (ISI) is a 7-item scale and is a widely used self-report measure of insomnia symptoms developed and validated by Bastien et al. [34]. It measures subjective symptoms, consequences, and distress associated with insomnia. Scores range from 0 to 28, with higher scores representing more insomnia. Currently, the ISI is scored such that scores between 0 and 7 represent no insomnia, scores from 8 to 14 represent subthreshold insomnia, scores from 15 to 21 represent moderate clinical insomnia, and scores from 22 to 28 represent severe clinical insomnia. Studies have found different optimal values for detecting clinically significant insomnia. A study by Gagnon et al. suggested that a cutoff score of 14 was found to be optimal for detecting clinical insomnia in a clinical sample [35]. Another study identified a cutoff score of 10 to be optimal for detecting insomnia in a community sample [36]. For ease of comparison to the existing literature, we examined frequency of insomnia symptoms based on the scoring of the measure and based on optimal cutoff scores identified by previous studies [35, 36]. In the present study, the ISI showed excellent reliability (Cronbach’s α = .91).

### Analysis

Statistical analyses were conducted with IBM SPSS Statistics, version 29.0.0.0 [37]. We evaluated the prevalence of sleep disturbance using descriptive statistics from the PSQI and ISI. We examined the prevalence of incarcerated women who had poor sleep quality (PSQI > 5), clinically significant insomnia (ISI > 14), and subthreshold insomnia (ISI > 7) using frequency statistics. We also assessed the prevalence of clinically significant depression and PTSD using frequency statistics. Independent samples *t*-tests evaluated if there were significant differences in sleep quality or insomnia severity by jail. We then ran two multivariate models, one with the PSQI total score as the dependent variable and one with the ISI total score as the dependent variable. Alpha was set to *p* < .05. Predictor variables included depression (CESD-R), PTSD symptoms (PCL-5), and trauma exposure (LSC-R). Age, race (coded 0 = white, 1 = nonwhite), and length of time in jail were entered as covariates since prior research suggests that these variables influence sleep [17, 38, 39]. The length of time in jail was log-transformed due to non-normality. Missing data were handled with listwise deletion. The percentage of missing values varied across study variables between 0% to 5.7%. There were missing values on the PSQI (*n* = 10), ISI (*n* = 4), LSC-R (*n* = 1), PCL-5 (*n* = 7) CESD-R (*n* = 6), and length of time in jail (*n* = 2).

## Results

### Descriptive statistics

Participants endorsed high rates of sleep difficulties. Nearly half of the women in the sample reported clinically significant insomnia symptoms (40.7% ISI > 14; 43.2% ISI ≥ 14; 58.5% ISI ≥ 10) on the ISI (*M *= 12.85, *SD* = 7.97, *n* = 172). A majority of the women (70.3%) reported subclinical threshold levels of insomnia symptoms (ISI > 7) or greater. Furthermore, 75.9% of participants presented with poor sleep quality (PSQI > 5; *M *= 9.20, *SD* = 4.21, *n* = 166). Women’s self-reported reasons for difficulty sleeping are summarized in Table 1. There were no differences between women in the two jails for insomnia symptom severity, *t*(170) = −0.57 *p* > .05, or sleep quality t(164) = 0.36, *p* > .05.

**Table 1.** Frequency Statistics for Sleep Variables (*N* = 176)

Variable	Mean (*SD*)
Insomnia Severity Index	12.85 (7.97)
Pittsburgh Sleep Quality Index	9.2 (4.21)
Sleep characteristics	***n* (%)**
Sleep quality (*n* = 166)	
Good sleepers (PSQI ≤ 5)	40 (24.1)
Poor sleepers (PSQI > 5)	126 (75.9)
Insomnia symptoms (*n* = 172)	
Absence of insomnia symptoms (ISI 0–7)	51 (29.7)
Subthreshold insomnia symptoms (ISI 8–14)	51 (29.7)
Moderate insomnia symptoms (ISI 15–21)	38 (22.1)
Severe insomnia symptoms (ISI 22–28)	32 (18.6)
Sleep duration (hours; *n* = 174)	
>11	9 (5.2)
10–11	11 (6.3)
9–10	9 (5.2)
7–9	44 (25.3)
6–7	31 (17.8)
5–6	26 (14.9)
< 5	44 (25.3)
Reported reasons for trouble sleeping (*n* = 172)	
Excessive noise	31 (18.0)
Inadequate or uncomfortable bedding	8 (4.7)
Lights	4 (2.3)
Mental health symptoms	40 (23.3)
Withdrawal from substances	4 (2.3)
Access to medication	1 (0.6)
Restless leg/ leg pain	4 (2.3)
Other	6 (3.5)

ISI, Insomnia Severity Index Total Score; PSQI, Pittsburgh Sleep Quality Index.

On average, women’s scores on the LSC-R indicated that they experienced high rates of trauma exposure (*M = *55.71, *SD* = 26.49, *n* = 175). Experiences of interpersonal trauma (i.e. physical or sexual assault) were reported by 87.4% of the sample, with over half of participants reporting that they experienced interpersonal trauma more than 10 times (Table 2). The women reported high levels of both PTSD and depressive symptoms. Around half (50.9%) of the sample exceeded the cutoff for clinically significant levels of PTSD symptoms on the PCL-5 (*M *= 34.21, *SD* = 18.87, *n* = 169), and 57.1% of the sample exceeded the cutoff for clinically significant levels of depression symptoms on the CESD-R (*M *= 20.08, *SD* = 13.20, *n* = 170).

**Table 2.** Prevalence of Criterion A Traumatic Experiences Reported on the LSC-R

Trauma variables	Mean (*SD*)
LSC-R	55.71 (26.49)
PCL-5	34.21 (18.87)
**Type of traumatic experience**	***n* (%)**
Lifetime interpersonal violence (physical or sexual assault; *n* = 174)	152 (87.4)
Lifetime physical abuse/assault (*n* = 175)	142 (81.1)
Physically abused/attacked as an adult (age ≥16) by a known perpetrator (*n* = 176)	127 (72.2)
Physically abused/attacked as a child (under age 16) by a known perpetrator (*n* = 176)	103 (58.5)
Robbed, mugged, or physically attacked by a stranger (*n* = 175)	69 (39.4)
Lifetime sexual abuse/assault (*n* = 175)	124 (70.9)
Sexual abuse/assault as a child (*n* = 175)	102 (58.3)
Sexual abuse/assault as an adult (*n* = 176)	94 (53.4)
Serious disaster (e.g. natural disaster, fire, explosion; *n* = 176)	61 (34.7)
Accident/accident-related injury (*n* = 176)	128 (72.7)
Witnessed a serious accident (*n* = 176)	122 (69.3)
Sudden or unexpected death of a close relative/friend (*n* = 175)	150 (85.7)
Witnessed violence between family members as a child (*n* = 175)	124 (70.9)
Witnessed a physical attack (e.g. robbery, mugging; *n* = 175)	90 (51.4)

LSC-R, Life Stressors Checklist-Revised total score; PCL-5, Posttraumatic Stress Disorder Checklist DSM-5 total score.

### Predictors of poor sleep quality and insomnia symptoms

As expected, depressive symptoms, trauma frequency, and PTSD symptoms were all significantly related to insomnia symptoms and poor sleep quality (Table 3). These univariate Pearson correlations indicated that as depressive symptoms, trauma frequency, and PTSD symptoms increased, insomnia symptom severity and poor sleep quality increased. The length of time in jail was related to sleep quality and insomnia symptoms. As the length of time in jail increased, insomnia symptoms and poor sleep quality decreased. Race was also related to sleep quality, with white individuals having better sleep quality than nonwhite individuals. In our first multiple regression, we regressed depressive symptoms, PTSD symptoms, and trauma frequency, on insomnia symptoms, with age, race (white = 0, nonwhite = 1), and length of time in jail as covariates. The overall regression was significant, *F*(6, 155) = 19.379, *p < *.001, *R*^2^ = 0.429, *R*^2^_adjusted_ = 0.406. Depression and PTSD were significant predictors of insomnia symptoms. The relations between PTSD and depression were in the expected direction, with greater PTSD and depressive symptoms predicting greater insomnia symptom severity. The results are presented in Table 4.

**Table 3.** Correlation Matrix Between Main Variables and Covariates

Variables	ISI	PSQI	PCL-5	LCS-R	CESD-R	Age	Race	Length_log_
ISI	—							
PSQI	0.702**	—						
PCL-5	0.461 **	0.426 **	—					
LCS-R	0.171*	0.247**	0.410**	—				
CESD-R	0.614**	0.541**	0.498**	0.263**	—			
Age	−0.001	0.018	−0.131	−0.094	0.013	—		
Race	−0.078	−0.168*	−0.106	−0.025	−0.185**	−0.163*	—	
Length_log_	−0.266**	−0.188*	−0.262**	−0.127	−0.237**	0.066	0.057	—

ISI, Insomnia Severity Index Total Score; PSQI, Pittsburgh Sleep Quality Index Total Score; PCL-5, Posttraumatic Stress Disorder Checklist DSM-5 Score with sleep Items removed; LCS-R, Life Stressors Checklist-Revised total score; CESD-R, Center for Epidemiological Studies Depression Scale Revised total score with sleep items removed; Length_log_, Log transformed number of days in jail; Race was coded as a dichotomous variable with white = 0 and nonwhite = 1. Correlations between two continuous variables are given as Pearson’s *r*. * signifies correlation is significant at *p* < .05 (two-tailed), ** signifies correlation is significant at *p* < .01 (two-tailed).

**Table 4.** Multiple Regression Models with Insomnia Sleep Quality as Outcomes

Outcome	Insomnia Severity Index			
Variable	Unstandardized *β* (SEM)	95%CI	Standardized *β*	*P* value
*(Constant)*	*6.850 (3.193)*	*0.543, 13.157*		*.033*
*PCL-5*	*0.106 (0.034)*	*0.040, 0.173*	*0.240*	*.002*
LCS-R	−0.032 (0.021)	−0.073, 0.010	−0.101	.131
*CESD-R*	*0.281 (0.042)*	*0.198, 0.363*	*0.486*	*<.001*
Age	0.046 (0.058)	−0.068, 0.160	0.049	.429
Race	0.492 (1.027)	−1.536, 2.519	0.030	.633
Length_log_	−2.092 (1.093)	−4.250, 0.067	−0.122	.057
Outcome	Pittsburgh Sleep Quality Index			
Variable	Unstandardized *β* (SEM)	95%CI	Standardized *β*	*P* value
(Constant)	*5.542 (1.854)*	*1.878, 9.206*		*.003*
*PCL-5*	*0.054 (0.020)*	*0.015, 0.093*	*0.231*	*.007*
LCS-R	−0.003 (0.012)	−0.028, 0.021	−0.020	.784
*CESD-R*	*0.128 (0.025)*	*0.079, 0.177*	*0.412*	*<.001*
Age	0.002 (0.033)	−0.064, 0.068	0.004	.950
Race	−0.466 (0.597)	−1.646, 0.714	−0.054	.437
Length_log_	−0.309 (0.631)	−1.555, 0.938	−0.034	.625

PCL-5, Posttraumatic Stress Disorder Checklist DSM-5 total score with sleep items removed; LCS-R, Life Stressors Checklist-Revised total score; CESD-R, Center for Epidemiological Studies Sepression Scale Revised total score with sleep items removed; Length_log_, log-transformed number of days in jail; race was coded as a dichotomous variable with white = 0 and nonwhite = 1. Items significant at *p* < .05 are in italics.

Next, depressive symptoms, PTSD symptoms, and trauma frequency were regressed on sleep quality, with age, race (white = 0, nonwhite = 1), and length of time in jail as covariates. This overall regression was also significant, *F*(6, 149) = 12.843, *p < *.001, *R*^2^ = 0.341, *R*^2^_adjusted_ = 0.314. Once again, depression and PTSD symptoms were significant predictors of sleep quality. These relations were in the expected direction; as depression and PTSD symptoms increased, sleep quality decreased. These results are presented in Table 4. Neither age nor race were significantly related to insomnia symptoms or poor sleep quality in the multiple regression models.

## Discussion

### Main findings

This is the first study to estimate the prevalence of sleep disturbance and related factors among incarcerated women jailed in rural Idaho. Our study found that insomnia symptoms, short sleep duration, and poor sleep quality were common in women in jail. Symptoms of PTSD and depression were associated with higher rates of poor sleep quality and insomnia symptoms.

Our sample had a high prevalence of clinically significant insomnia symptoms (40.7% ISI > 14; 58.5% ISI > 10). In contrast, previous research suggests that approximately 10% of the general population has clinically significant insomnia [40]. Similar to previous studies, our results suggest women incarcerated in jail experience much greater levels of insomnia symptoms than the general population [5]. Participants also reported high rates of poor sleep quality (75.9%). These rates of poor sleep quality are much higher than those of the general population, as previous studies have estimated that 25% to 36% of individuals in the general population have poor sleep quality [5, 11, 13]. Just over 40% of the women in our sample reported sleeping less than 6 hours per night. Recent figures from the National Sleep Foundation estimate that approximately one-third of adults sleep six or fewer hours per night [41]. It would appear that incarcerated women also experience higher rates of short sleep than the general population. Future studies should compare these levels with inferential statistics.

It is important to note that the literature in this area has generally focused on prison populations. In contrast, we assessed sleep quality in women in jails, which tend to be more crowded and have higher inmate turnover than prisons. However, our results are reasonably consistent with studies that have examined poor sleep quality and insomnia in other incarcerated populations. A recent review article noted that incarcerated individuals experience high levels of poor sleep quality and insomnia [5]. This article included sleep disturbance results from nine countries and twelve incarceration settings. In each country and setting, rates of poor sleep quality and insomnia were high compared to those experienced in the general population. However, the rates of insomnia and poor sleep quality varied quite a bit across settings. These variations suggest that it is important to consider the contextual factors that may impact how well individuals sleep while incarcerated. For instance, we need to consider the country of origin, incarceration setting (short-term vs. long-term), environmental factors of each correctional facility, and sex of inmates. Our findings add to the literature on sleep and incarceration by illustrating that sleep disturbance occurs at high levels amongst women in short-term incarceration settings (e.g. jails) in rural United States.

These findings indicate that even short-term incarceration may have detrimental effects on women’s sleep. There are several important implications of this finding. Prior research demonstrates that sleep disturbance has a negative impact on physical health [4]. Thus, sleep disturbance experienced during incarceration may negatively impact physical health. Studies also show that individuals with poor health are overrepresented in correctional facilities [42]. Thus, the sleep disturbance they experience due to incarceration may exacerbate preexisting health disparities. We must better understand the factors contributing to sleep problems in this vulnerable population to inform planning and offer suggestions to improve correctional environments.

In our study, we examined trauma exposure and mental health as predictors of sleep disturbance in incarcerated women. As anticipated, higher PTSD and depressive symptoms both correlated to greater insomnia symptom severity and poorer sleep quality. A previous study that examined correlates of sleep problems in Chinese prisoners found that depression and PTSD were highly predictive of insomnia in prison [43]. These results demonstrate that individuals with PTSD and depression may be more likely to experience sleep disturbance while incarcerated. Correctional facilities could consider screening for these conditions and offering programming to help mitigate the impact of PTSD and depression on sleep.

We also examined the relationship between lifetime exposure to trauma, insomnia symptoms, and poor sleep quality. While lifetime exposure to trauma correlated with insomnia symptoms and sleep quality, trauma exposure was not a significant predictor of sleep quality or insomnia symptoms in the full regression models. It is likely that symptoms of posttraumatic stress and depression mediate the relation between lifetime exposure to trauma and sleep. In the context of the 3P model of insomnia [15] this suggests that trauma exposure is a precipitating factor for the development of insomnia in these women, and that behavioral symptoms of PTSD (e.g. behavioral avoidance and hypervigilance) continue to perpetuate insomnia symptoms after the acute stressor of trauma exposure dissipates. Future research should employ directional methods (e.g. longitudinal methods) to evaluate this mechanism. Further research may also consider investigating the relation between specific types of trauma (e.g. interpersonal violence or sexual violence) and sleep. This relationship may be dependent on the type of trauma given that exposure to interpersonal trauma tends to be associated with worse mental health outcomes in general, and sexual violence in particular is related to worse mental health outcomes for women [44, 45].

Another thing to consider is the clinical implications of the bidirectional nature of the associations between PTSD and sleep and depression and sleep [27]. It is possible that sleep disturbance caused by other factors in correctional facilities may contribute to higher PTSD and depression symptoms. Given this interpretation, future research should examine the impact of environmental changes targeted at improving sleep conditions or sleep-specific programming. Researchers might consider adapting the gold standard treatment for insomnia, Cognitive Behavioral Therapy for Insomnia, to incarcerated populations [46]. These interventions may improve sleep. They may also indirectly improve PTSD and depressive symptoms that are made worse by poor sleep.

Regarding other factors that impact sleep, participants indicated that they had trouble sleeping in jail due to excessive noise, inadequate or uncomfortable bedding, lights, mental health symptoms, or withdrawal from substances. We did not directly ask whether specific environmental factors impacted participants’ ability to sleep, and it is likely that even more women were affected by these environmental factors than our results indicate. Regardless, it appears that both environmental and individual factors are detrimental to healthy sleep in this population.

These findings are consistent with the lawsuits against correctional facilities for environmental conditions that negatively impact sleep [10]. These results indicate that environmental factors exacerbated in the incarceration environment influence sleep quality. Correctional facilities have an ethical and legal responsibility to provide humane treatment to detained individuals. Intentional sleep deprivation has been denounced internationally as a form of cruel and unusual punishment [47]. Corrections staff must be made aware of how these conditions impact inmates’ sleep and the potential impact of sleep deprivation on women’s health. Future research should examine which environmental factors significantly predict poorer sleep outcomes in correctional facilities. This information would help correctional facilities to change environmental factors leading to sleep deprivation and likely lead to better physical and mental health outcomes for incarcerated individuals.

## Limitations

Several limitations to this study should be considered when interpreting the results. Prior studies have found sex differences in insomnia symptoms [9], so results may not generalize to males incarcerated in jail. This study was conducted cross-sectionally. This means we cannot determine if incarceration leads to sleep difficulties, which increase rates of depression and PTSD, or if incarceration increases PTSD and depression, which cause increased sleep disturbance, or if incarcerated individuals are simply at risk for higher levels of PTSD, depression, and sleep disturbance. The results are also limited because we did not have a formal demographically matched comparison group for our study sample, so we cannot determine if our sample of incarcerated women is significantly different than the general population. The reliability of the PSQI was only acceptable in this study. This is likely due to the sleep medication item because women struggle to access sleep medication in jail. When this item was dropped from the Cronbach’s alpha calculation, reliability increased to 0.74. We decided to include the full questionnaire in our analyses to interpret poor sleep quality based on established cutoffs. However, the reliability of this measure in our study sample is a limitation of our study. Lastly, our study relied on self-report measures, which are subject to recall bias.

## Conclusions

This study suggests that insomnia symptoms and poor sleep quality are common among women incarcerated in jails in rural Idaho. PTSD symptoms and depressive symptoms were associated with insomnia symptoms and sleep quality, such that higher rates of PTSD and depression symptoms correlated with higher insomnia symptoms and poorer sleep quality. We also identified several environmental factors (noise, light, access to medication, and poor bedding) that women reported impacted their ability to sleep in jail. Future studies should examine these environmental factors to see if they predict sleep outcomes. Additionally, given the high rates of PTSD symptoms, depression symptoms, and insomnia symptoms, programming targeting these concerns would likely improve inmate health outcomes. Given the high mortality rates for previously incarcerated women [1, 2], it is critical that we identify potential points of intervention to improve health outcomes for this vulnerable population.

## Data Availability

The data from this study is not publicly available at this time but is available from the corresponding author on reasonable request.

## Notes

This paper is part of the *Sleep and Circadian Health in the Justice* System Collection.
